# Effectiveness, Safety, and Real-World Experience of Brolucizumab: A Systematic Review

**DOI:** 10.3390/ph18111620

**Published:** 2025-10-27

**Authors:** Naif M. Alali, Abeer Aljahdali, Hani B. AlBalawi, Othman Jarallah Al Jarallah, Salem Mohammed AL Zaid, Ismail Abuallut, Faisal ALMarek, Ibrahim Shajry, Yousef A. Alotaibi, Mohammad A. Hazzazi, Moustafa S. Magliyah

**Affiliations:** 1 Division of Ophthalmology, Department of Surgery, Faculty of Medicine, University of Tabuk, Tabuk 71491, Saudi Arabia; nmalali@ut.edu.sa; 2 Vitreoretinal Division, King Khaled Eye Specialist Hospital, Riyadh 11462, Saudi Arabiamussam8@yahoo.com (M.S.M.); 3 Ophthalmology Department, King Abdulaziz University, Jeddah 21589, Saudi Arabia; 4 Department of Ophthalmology, AdDiriyah Hospital, Third Health Cluster, Riyadh 13717, Saudi Arabia; 5 Division of Ophthalmology, Department of Surgery, Faculty of Medicine, Jazan University, Jazan 45142, Saudi Arabia; 6 Faculty of Medicine, Imam Mohammed Ibn Saud Islamic University, Riyadh 11432, Saudi Arabia; 7 King Khalid University Medical City, King Khalid University, Abha 61421, Saudi Arabia; 8 Department of Ophthalmology, College of Medicine, King Khalid University, Abha 61421, Saudi Arabia; 9 Department of Ophthalmology, King Abdulaziz Medical City, Riyadh 11426, Saudi Arabia

**Keywords:** brolucizumab, efficacy, safety, pharmacokinetics, real-world outcomes, cost-effectiveness

## Abstract

**Background/Objectives:** Brolucizumab is a humanized single-chain antibody fragment with a molecular weight of approximately 26 kilodaltons (scFv, ~26 kDa) targeting all VEGF-A isoforms. Intravitreal brolucizumab (6 mg) is FDA-approved for neovascular age-related macular degeneration (nAMD) (2019) and diabetic macular edema (DME) (2022). We systematically review the literature on brolucizumab for nAMD and DME, focusing on efficacy, safety, pharmacokinetics, real-world outcomes, and cost-effectiveness in adult and pediatric patients. **Methods:** Our method involves a comprehensive literature search of PubMed, Embase, Scopus, Cochrane, and related databases (through late 2024) using terms including “brolucizumab,” “Beovu,” “neovascular AMD,” “diabetic macular edema,” “safety,” “pharmacokinetics,” and “pediatric.” High-quality clinical trials, meta-analyses, regulatory documents, and real-world studies were prioritized. **Results:** In pivotal Phase III trials (HAWK/HARRIER for nAMD), brolucizumab 6 mg demonstrated non-inferior visual acuity (VA) gains to aflibercept, with >50% of eyes maintained on 12-week dosing and greater retinal fluid reduction. In DME trials (KESTREL/KITE), brolucizumab was similarly non-inferior to aflibercept for VA and showed superior anatomic drying, with 33–48% of eyes maintained on ≥12-week intervals. However, brolucizumab use has been associated with intraocular inflammation (IOI), retinal vasculitis, and vascular occlusion: clinical trials and post hoc analyses reported higher rates of these events than comparator agents. Real-world cohorts found IOI in ~4–10% of treated eyes, often occurring early (within 3 months) after initiation. **Conclusions:** In conclusion, Brolucizumab is an effective anti-VEGF option for nAMD and DME, providing durable anatomic control with fewer injections. Non-inferior vision outcomes and superior fluid resolution have been demonstrated. However, it carries a distinct risk of IOI and occlusive vasculitis, necessitating careful patient selection, dosing, and monitoring.

## 1. Introduction

Neovascular (“wet”) age-related macular degeneration (nAMD) and diabetic macular edema (DME) are the most common adult causes of vision loss. nAMD is caused by choroidal neovascularization due to vascular endothelial growth factor (VEGF) overexpression, leading to retinal exudation and fibrosis [1,2]. DME results from diabetic retinal vascular leakage and VEGF-A upregulation, leading to macular edema and vision loss [3,4]. In both diseases, standard therapy is intravitreal anti-VEGF, which significantly improves outcomes compared to previous therapies [5,6,7]. However, frequent injections (typically monthly) are a burden to patients and health systems. Consequently, agents that are both more durable and effective are needed.

Brolucizumab (Beovu^®^) is an innovative anti-VEGF molecule licensed by the FDA for nAMD (October 2019) and DME (June 2022) [8]. It is a humanized single-chain antibody fragment (scFv) that binds VEGF-A isoforms [9]. At ~26 kDa, it is considerably smaller than ranibizumab (48 kDa) and aflibercept (115 kDa). Being small, together with high solubility, high molar dosing (6 mg/0.05 mL) becomes possible, retaining more VEGF-binding capability/molecule per injection, potentially allowing longer dosing intervals [9,10]. Pivotal studies indicated up to 50% of eyes treated with brolucizumab would be eligible to be retreated on a 12-week basis during year one [11].

In contrast to the promise for less frequent treatment, the safety profile of brolucizumab differed from predecessors. Spontaneous post-marketing reports recognized instances of serious intraocular inflammation (IOI), retinal vasculitis, and occlusive vasculopathy resulting in vision loss [12,13,14,15,16]. This led to label revisions and warning. However, numerous eyes are helped by brolucizumab, and real-world data are being introduced. Moreover, pharmacokinetic, immunogenicity, and economic factors must be taken into consideration. Therefore, we performed a systematic review of brolucizumab for nAMD and DME, grounded on clinical research, meta-analyses, real-world studies, and regulative data. Herein, we outline the pharmacology, trial effectiveness, safety indicators, observational outcomes, pediatric concerns, and the cost-effectiveness.

## 2. Methods

### 2.1. Review Design and Eligibility Criteria

The protocol was developed a priori using the PECOS framework and was implemented and reported in accordance with PRISMA 2020 reporting guidelines [17]. The protocol was registered in the PROSPERO database (CRD420251157792). Population (P): Individuals with neovascular age-related macular degeneration (nAMD/wet AMD) or diabetic macular edema (DME) who received intravitreal therapy in randomized or observational settings; pediatric populations were considered when data existed. Exposure/Intervention (E): Intravitreal brolucizumab (Beovu^®^), any approved or investigational dose, initiation strategy (fixed, pro re nata, or treat-and-extend), and switching sequences; pharmacokinetic/pharmacodynamic (PK/PD) and device-related administration details were recorded when reported. Comparator (C): Active anti-VEGF comparators (e.g., aflibercept, ranibizumab, bevacizumab), alternative dosing strategies of brolucizumab, or historical/parallel cohorts in real-world studies; single-arm evidence was retained for safety, PK/PD, and utilization outcomes. Outcomes (O): Effectiveness (best-corrected visual acuity change in ETDRS letters; ≥10/≥15-letter gain/loss; central retinal/subfield thickness; anatomic drying [IRF/SRF resolution]; fluid status; treatment interval extension; injection frequency), safety (any adverse event; intraocular inflammation; retinal vasculitis; retinal vascular occlusion; endophthalmitis; discontinuations), PK/PD (ocular/systemic exposure, time to Cmax, AUC, target engagement), and health-economic/real-world metrics (persistence, switching, visits, costs, cost-effectiveness). Study design/Setting (S): Randomized controlled trials (parallel-group or cross-over), non-randomized comparative studies, prospective or retrospective cohorts, large case series, regulatory documents (FDA/EMA labeling and assessment reports), and health technology assessment summaries; multi-country and single-center settings were included without date limits and restricted to English-language sources.

### 2.2. Inclusion and Exclusion Criteria

Eligibility criteria were established a priori and used during two-stage screening (titles/abstracts, then full text) by two independent reviewers with consensus resolution. Studies were included if they included patients with nAMD or DME treated with intravitreal brolucizumab; presented a minimum prespecified effectiveness, safety, PK/PD, economic, or real-world utilization outcome; and used randomized, non-randomized comparative, cohort, or large case-series designs. Regulatory documents (FDA/EMA labels, as well as assessment reports) and health technology assessment yielding structured evidence on safety, PK/PD, or effectiveness were included as contextual evidence. English-language records without the restriction on date of publication were eligible. Studies were excluded if they were non-human, in vitro, or animal research; narrative reviews, editorial comments, letters (without extractable data), conference abstracts (without full report), case report(s) or case series involving <10 participants/arm/timepoint, mixed-indication reports without stratified nAMD/DME data, or studies where there was insufficient information to extract prespecified outcomes despite attempts to contact authors. Duplicates and overlapping datasets were recognized through the use of bibliographic management, as well as cross-checking; the most comprehensive, non-overlapping dataset was kept.

### 2.3. Database Searching Procedure

Sources included PubMed/MEDLINE, Embase (Elsevier), Scopus (Elsevier), the Cochrane Library (CENTRAL and Cochrane Reviews, Clarivate), and Google Scholar. We used controlled vocabulary (MeSH/Emtree) as well as free-text synonyms for the molecule (brolucizumab/Beovu), target indications (nAMD/wet AMD; diabetic macular edema), and concepts related to the evidence (safety, intraocular inflammation, PK/PD, real-world, pediatric, cost-effectiveness). Truncation, proximity, and field tags were tailored to each database. No date restrictions were applied; English-language filters, along with human-study limits, were applied where local holdings existed (Table 1). Key article references as well as those from regulatory/HTA documents were hand-searched. De-duping was carried out in a reference manager before screening.

### 2.4. Data Extraction Strategy and Data Items

Data extraction was performed separately by two reviewers who used a piloted, standardized form; any conflict was resolved by consensus or third-party evaluation. Study-level items captured bibliographic details; funding/conflicts; research design (RCT, non-randomized comparative, cohort, case-series), setting (country, center type), enrolment period, and sample size. Participant-level items captured indication (nAMD/DME), age, sex, baseline best-corrected visual acuity, baseline central retinal/subfield thickness, lesion features (type, size), duration of disease, former anti-VEGF exposure, laterality, and significant comorbidities (e.g., duration of diabetes for DME). Intervention items captured brolucizumab dose, loading/maintenance regimen (fixed/PRN/treat-and-extend), switching threshold, concomitant ocular therapy, and procedure aspects. Comparator items captured comparator agent, dose, and regimen or other brolucizumab regimens. Effectiveness outcomes captured mean change in ETDRS letters, proportions achieving ≥10/≥15-letter gain/loss, change in central thickness, proportions achieving anatomic drying/fluid resolution, disease activity, injection interval attained, and number of injections/visits annually. Safety outcomes captured adversities (any/serious), intraocular inflammation, retinal vasculitis, retinal vascular occlusion, endophthalmitis, systemic event(s) of interest, discontinuations, and time-to-event where captured. PK/PD items captured concentrations (ocular/systemic), Cmax, Tmax, AUC, half-life, as well as target engagement markers. Economic/real-world items captured persistence, adherence, switching behavior, resources utilized, direct costs, and incremental cost-effectiveness ratios. Extraction captured analysis population definitions, missing data handling, adjustment for covariates, effect measures including precision (corresponding associated confidence intervals), and follow-up periods. Where multiple reports presented identical cohorts, the most extensive dataset was kept, reviewed, and checked to avoid double-counting.

### 2.5. Bias Assessment Protocol

Risk of bias was rated independently by two reviewers, with agreement resolution. In randomized trials, the Cochrane RoB 2.0 instrument [18] was used across the following five domains (randomization process; deviations from planned interventions; missing outcome data; measurement of the outcome; selection of the reported result) to produce domain-level and overall judgments (low risk, some concerns, or high risk) that included recorded justifications. In non-randomized studies, ROBINS-I [19] was used across seven domains (confounding; participant selection; classification of the intervention; deviations from planned interventions; missing data; measurement of outcomes; selection of the reported result) to produce overall judgments (low, moderate, serious, or critical risk bias). Prespecified confounders were baseline visual acuity, central thickness, lesion subtype, duration of the disease, previous exposure to anti-VEGF, bilaterally of the disease, and clinic-level treatment policies. Risk-of-bias results were used to qualify narrative synthesis and to guide sensitivity considerations.

## 3. Results

**Figure 1:**
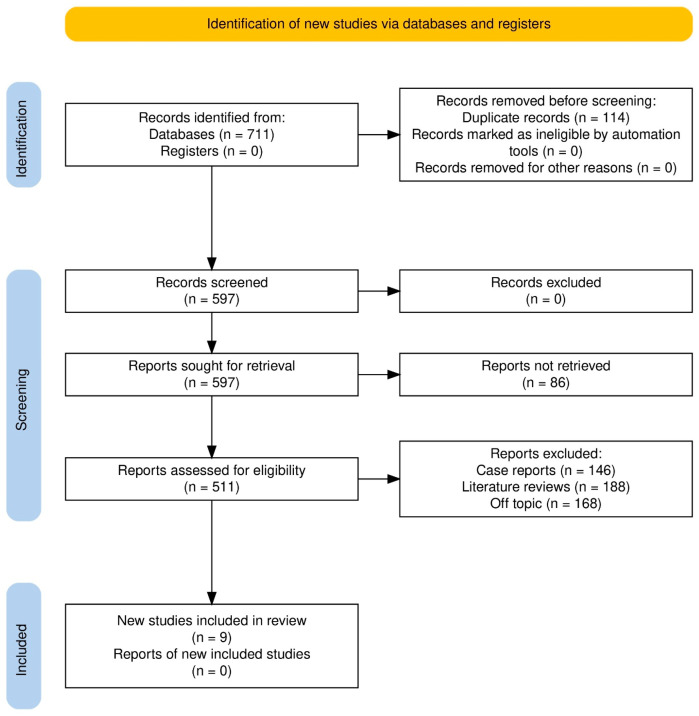
Study selection process.

We collected 711 records from the databases and 0 from registers (Figure 1); 114 duplicates were excluded before title/abstract screening, leaving us with the 597 original records (Figure 1). Title/abstract screening excluded no records, so retrieval of all 597 reports was attempted; 86 were unavailable. Full-text eligibility assessment was performed on 511 reports, whereupon 502 were excluded (*n* = 146 case reports, *n* = 188 literature reviews, *n* = 168 off-topic). We included nine studies [11,12,20,21,22,23,24,25,26], and none of the other included studies’ reports were found.

### 3.1. Bias Levels Observed

**Figure 2:**
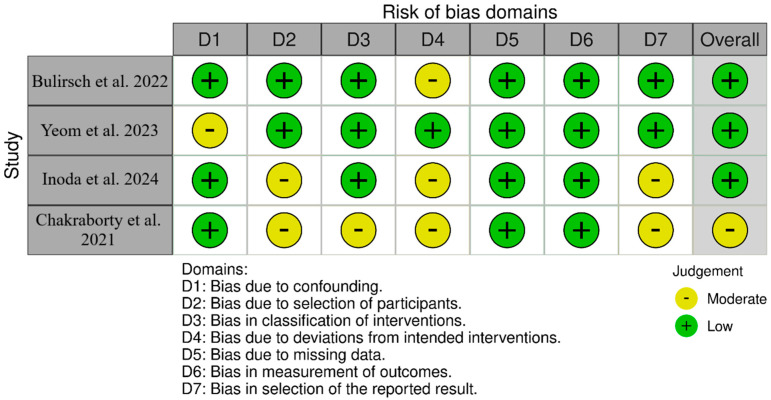
Bias assessment across the included cohort studies [20,21,22,23].

**Figure 3:**
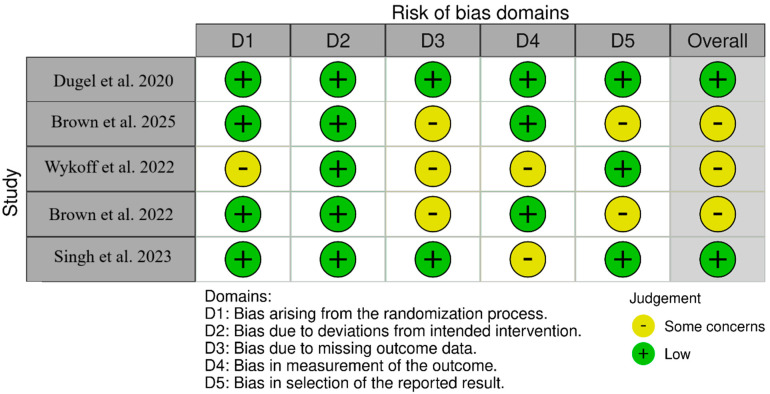
Bias assessment across the included RCTs [11,12,24,25,26].

For non-randomized studies (ROBINS-I), as shown in Figure 2, SHIFT [20] had low overall risk with moderate concerns about deviations from intended interventions (D4). Yeom et al. [21] had low overall risk with moderate concerns about confounding (D1) and selection of the reported result (D7). Inoda et al. [22] had low overall risk with moderate concerns about selection of participants (D2) and deviations (D4). BRAILLE [23] had moderate overall risk due to moderate concerns in D2, D3, D4, and D7. For randomized trials (RoB 2-Figure 3), HAWK [11] was low-risk across all; HARRIER [11] had some concerns due to randomization (D1) and measurement (D4); MERLIN [12] had some concerns due to missing outcome data (D3) and selective reporting (D5); KESTREL [24] and KITE [24,25] each had some concerns across the domains of randomization and measurement; and KINGFISHER [26] had low overall risk with some isolated concerns regarding measurement (D4).

### 3.2. Demographic Variables Assessed

In both randomized and real-world studies, participant nAMD groups were older (mean age ~76 years old in pivotal RCTs) and mostly treatment-naïve in Phase III studies, while real-world groups were mostly switch or refractory populations, demonstrating clinical heterogeneity and an extended duration of the disease [11,20,21,22,23] (Table 2). Trials’ follow-up lasted up to 96–100 weeks, allowing durability evaluation, while observational cases routinely provided 52-week results with regimen flexibility (PRN or T&E) [11,22,23,24,25]. Baseline functional and anatomic measures were inconsistently presented among the summaries offered; nevertheless, the baseline high fluid burdens among the refractory cohort populations, as well as previous exposure to anti-VEGF therapies, signaled advanced disease that limited short-term functional improvement despite anatomic gains [20,21]. Long, multinational cohort sizes were common among the DME trials, and KINGFISHER included both naïve as well as previously treated patients on an interval-matched monthly schedule [24,25,26].

### 3.3. Outcomes and Parameters Assessed

In key nAMD trials, brolucizumab allowed for q12-week maintenance in approximately half of the eyes after loading, equivalent to aflibercept on BCVA, and provided superior drying and the largest CST reductions by week 48, allowing decreased burden without vision compromise [11] (Table 3). Extended follow-up upheld durability and indicated a preferential advantage in fluid-refractory phenotypes [19]. Monthly dosing in MERLIN enhanced anatomic endpoints but elevated IOI and vascular event rates compared to aflibercept, affirming ≥q8-week intervals following loading [12]. Real-world switch arms verified high anatomic response rates, significant interval extension (often to ~12–13 weeks), modest-but-clinically significant BCVA improvements in less damaged retinas, and low injection numbers under PRN regimens, indicating effective utilization in refractory practice paradigms [20,21,22,23]. In DME, KESTREL/KITE confirmed non-inferior BCVA with more frequent 12-week intervals and improved fluid control vs. aflibercept to 100 wks, while the interval-matched KINGFISHER trial revealed larger CST reductions, higher fluid-free rates with monthly brolucizumab, and a safety profile aligned with recognized inflammatory danger at higher regimens [24,25,26].

### 3.4. Evidence Map

**Figure 4:**
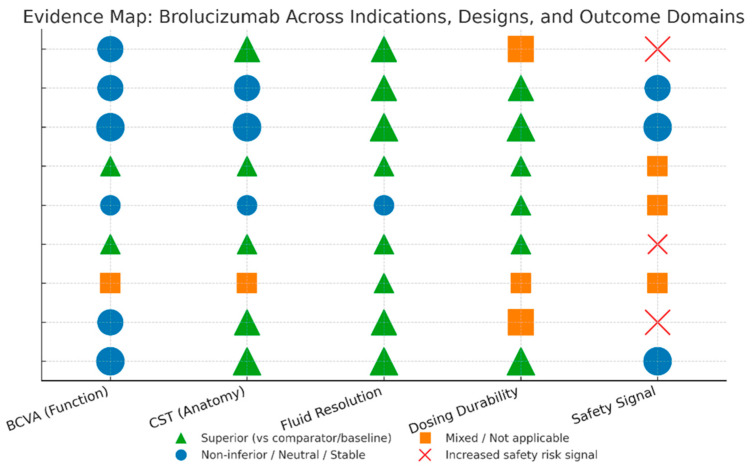
Evidence map visualization.

Throughout treatment-naïve nAMD, HAWK/HARRIER demonstrated non-inferior BCVA with superior drying and higher CST reductions, and about half of the eyes retained q12-week dosing after loading, validating lower treatment burden without vision compromise [11] (Figure 4). The monthly MERLIN regimen enhanced anatomy as well as fluid-free status but presented an unmistakable safety signal (IOI, vasculitis/occlusion), affirming ≥q8-week intervals beyond loading [12]. Real-world switch data (SHIFT; Yeom; Inoda; BRAILLE) invariably substantiated strong fluid control as well as manageable interval extension (often to ~12–13 weeks), with functional improvements most notable where irreversible harm was less progressed; an IOI signal indicating acknowledged risks was observed in one cohort, while various series did report safety events explicitly [20,21,22,23].

In DME, KESTREL/KITE demonstrated non-inferior BCVA with superior fluid control and substantial q12-week maintenance up to 100 weeks, demonstrating long-lasting efficacy with shorter visit intervals than fixated q8-week comparators [24,25]. In the interval-matched monthly KINGFISHER trial, the former results were verified, achieving wider CST reductions and higher fluid-free rates, together with a slightly elevated inflammatory signal, as expected at the higher doses [26].

## 4. Discussion

A recent systematic review and meta-analysis of brolucizumab in DME and diabetic retinopathy found that brolucizumab significantly improved BCVA and central subfield macular thickness (CSMT) relative to comparator anti-VEGFs [27]. The pooled mean differences favored brolucizumab: MD −0.64 (95% CI −1.15 to −0.13) in logMAR BCVA (*p* = 0.01) and MD −138.6 µm (95% CI −151.9 to −125.3) in CSMT (*p* < 0.00001). The authors concluded that brolucizumab offers potential efficacy advantages (visual gain, retinal drying, and extended dosing intervals) over other agents in DME/DR. However, they cautioned that sight-threatening adverse events occurred more frequently with brolucizumab (notably retinal vasculitis), especially at the 3 mg dose (no randomized comparisons were made between different brolucizumab doses). Overall, the evidence supports the effectiveness of brolucizumab for DME, consistent with its regulatory approval for use in adults.

In refractory DME, limited data exist. A small Portuguese series (59 eyes) showed that a single brolucizumab injection produced modest VA and OCT improvements at 1 month, especially in previously responsive cases [28]. In a short real-world study, Chakraborty D et al. investigated the efficacy of brolucizumab in treating patients with chronic diabetic macular edema who had not responded well to previous anti-VGEF therapies. The central retinal thickness and visual acuity of all 13 patients who completed the 12-week follow-up after the initial brolucizumab injection showed significant improvements. Approximately 92% of patients who had a 16-week follow-up after their first injection of brolucizumab were able to prolong their treatment course to 16 weeks. Importantly, throughout the follow-up, no occurrences of intraocular inflammation or other safety concerns were observed, suggesting that brolucizumab could be a good option in some refractory DME cases [29]. Longer-term real-world DME data are scarce, but its strong anti-VEGF activity supports the expectation that it may deliver sustained benefits over time. Anecdotal experience suggests similar effects as in nAMD: many patients maintain/improve vision with fewer injections.

Brolucizumab’s most notable safety concern is immune-mediated intraocular inflammation (IOI), including occlusive retinal vasculitis (RV) and retinal vascular occlusion (RO). In the pivotal HAWK/HARRIER trials, overall safety (all adverse events) was reported as generally similar between brolucizumab and aflibercept [11]. However, early post-approval reports signaled higher rates of IOI with brolucizumab than anticipated. Novartis initiated a review and the American Society of Retina Specialists (ASRS) issued alerts of occlusive vasculitis cases. The brolucizumab US prescribing information lists IOI occurring in ~4% of treated eyes and retinal artery occlusion in ~1% [30].

A meta-analysis of RCTs found no significant difference in all-cause adverse events between brolucizumab and aflibercept [5], but did find a higher odds of serious ocular adverse events with brolucizumab (OR 2.15 vs. aflibercept/ranibizumab; *p* = 0.02). Real-world studies provide a clearer picture. The IRIS Registry analysis is one of the largest real-world studies of brolucizumab safety outcomes. It looked at data from about 15,998 patients and 18,312 eyes that were treated for nAMD in the US. During the two years of follow-up, 3.4% of eyes had an intraocular inflammation (IOI), retinal vasculitis, or vascular occlusion event. Most of these events happened in the first six months of treatment [31]. In a US multicenter series (*n* = 482 eyes) of nAMD patients receiving brolucizumab, IOI-related adverse events occurred in 4.6% of eyes. Within these, 0.8% developed retinal vasculitis and 0.4% had retinal vascular occlusion. Most events (64%) manifested within the first 3 months of therapy. Notably, 3 of 22 eyes (14%) with IOI lost ≥30 ETDRS letters at event time, and 5 (1.0%) lost ≥15 letters; however, by 3–6 months after event resolution, the majority had recovered to within 5 letters of pre-event VA [32]. In the BRAILLE study, the IOI was 3.66%, and all patients had mild inflammation and were handled conservatively. Additionally, there were no documented cases of vascular occlusion or vasculitis [23]. Yeom et al. evaluated 81 patients with refractory nAMD who had not responded well to different anti-VEGF agents and were switched to brolucizumab. In their study, they discovered a higher rate of IOI (8.6%) and retinal vascular occlusion (1.2%) than in earlier published research [21]. In the SHIFT study, IOI occurred in 7 out of 63 treated eyes, representing 11.1% of the eyes that were followed up after 1 month. Considering that 207 intravitreal brolucizumab injections were given to the entire study, the IOI rate per injection was approximately 3.38%. The IOI included anterior uveitis, intermediate uveitis, and one case of non-occlusive retinal vasculitis. The treatment was tailored according to the severity of the inflammation. All patients were treated successfully and there was no permanent structural damage or significant drop of vision [20].

Given these findings, AAO expert consensus emphasizes that careful patient selection and monitoring are essential. Brolucizumab-treated eyes should be evaluated for inflammation at each visit. If IOI occurs, prompt corticosteroid therapy (topical, intravitreal, or systemic depending on severity) is recommended and treatment withheld [33]. Updated labeling (October 2020 FDA update) advises not to administer brolucizumab more frequently than 8-week intervals due to IOI risk [34]. The MERLIN trial reinforced this: eyes dosed every 4 weeks had significantly higher IOI (11.5% vs. 6.1% with aflibercept) [12], leading to early termination of that regimen. In the KINGFISHER trial, IOI occurred in 4.0% of brolucizumab-treated eyes versus 2.9% with aflibercept; uveitis (2.3%), retinal vasculitis (0.9%), and retinal vascular occlusion (0.3%) were the main events. Although both KINGFISHER and MERLIN used 4-week dosing, the IOI rate was notably lower in KINGFISHER for unclear reasons [26]. The retrospective Cleveland clinic cohort (*n* = 482) provided detailed safety and outcome data [32]. Overall, 418 patients received at least one brolucizumab injection; 4.6% developed IOI-related events. Importantly, outcomes suggest that with close follow-up, permanent visual loss can be minimized. After IOI, 82% of affected eyes maintained vision within five letters of baseline by 3–6 months. Only 14% of eyes with IOI had lost ≥5 letters at that time. The rate of new IOI declined with subsequent injections, implying a “vigilance washout” effect. Most hemorrhage or vascular occlusion occurred in eyes with IOI, underscoring the link between inflammation and vascular events. The authors concluded that early identification and management of IOI can preserve vision [32]. Overall, IOI and vasculitis are serious, albeit infrequent, adverse effects. Studies suggest that the incidence is lower in real-world practice when physicians adhere to on-label dosing intervals and maintain close monitoring for symptoms and signs of IOI [12,32].

Endophthalmitis and other injection-related complications occur at expected low rates (<0.1% per injection) and are not higher with brolucizumab than other intravitreal therapies. Reports of retinal detachment, traumatic injury, or cataract progression are anecdotal and not clearly linked to the drug. In HAWK/HARRIER, other ocular safety endpoints (e.g., intraocular pressure elevation) were similar between groups [11].

Systemic VEGF inhibition can theoretically increase risks of hypertension, thromboembolism, or proteinuria, but large ophthalmic anti-VEGF trials have not consistently shown these for intraocular dosing [8]. Brolucizumab’s low systemic exposure suggests even lower systemic risk. In HAWK/HARRIER, rates of arterial thromboembolic events (ATEs) were similar between brolucizumab and aflibercept arms [11]. No unexpected systemic safety signals (e.g., myocardial infarction, stroke) have emerged in the brolucizumab trials or real-world cohorts [21,23]. As with other intravitreal VEGF inhibitors, caution is advised in patients with recent stroke or myocardial infarction per labeling, but no formal contraindication exists.

Brolucizumab has not been studied in pediatric patients. The US label explicitly states that safety and efficacy in children have not been established [8]. Developmental and ocular anatomy differences make extrapolation from adults unreliable. We did not find clinical trials of brolucizumab in pediatric retinal diseases. Off-label use is limited but anecdotal. A case report describes a 9-year-old with Coats’ disease (a pediatric exudative retinopathy) who received intravitreal bevacizumab initially, then brolucizumab when fluid persisted [35]. The edema resolved completely after brolucizumab with no recurrence through 5 months. While intriguing, such isolated cases provide insufficient evidence for pediatric indication. Similarly, no formal reports exist for juvenile diabetic macular edema. Thus, current guidance is to avoid brolucizumab in children except in experimental or compassionate-use scenarios, with careful risk–benefit discussion.

The pharmacokinetic profile of brolucizumab helps explain its clinical effect. The high intraocular molar concentration achieved by 6 mg dosing means more VEGF binding in the retina, leading to potent and durable fluid control. The vitreous elimination half-life of brolucizumab is on the order of days to weeks (systemic t^1/2^ ~4.4 days) [8]. This underlies the possibility of 12-week (or longer) dosing. Clinically, this translates into many patients going 12 weeks or more without recurrence [11,19]. Pharmacodynamic studies (OCT volumetrics) have shown that brolucizumab more completely dries subretinal/intraretinal fluid compartments [10], reflecting its potency.

Immunogenicity is a PK/PD consideration. The high rate of anti-brolucizumab antibodies may contribute to the inflammation signal. These antibodies likely form immune complexes in the eye, triggering IOI/vasculitis [8]. No pre-treatment screening is standard [8]. Expert opinion advises choosing patients carefully, especially if they have a history of intraocular inflammation [34]. Furthermore, no IOI prophylactic has been approved [34].

Pharmacoeconomic analyses suggest that the greater durability of brolucizumab can translate into lower overall costs. In nAMD, models incorporating injection frequency, drug price, and outcomes have been performed. A Markov cost-utility analysis in Italy (15-year horizon, NHS perspective) found that brolucizumab *dominated* aflibercept (i.e., lower cost and higher quality-adjusted life years) [36]. The analysis reported savings of ~EUR 15,679 and a modest QALY gain (+0.11) with brolucizumab vs. aflibercept. In sensitivity analyses, brolucizumab remained cost-effective in ≥84% of simulations at a EUR 50,000/QALY threshold. The authors attributed savings to fewer injections and monitoring visits needed with brolucizumab.

In the United States, budget-impact models likewise projected net cost savings from brolucizumab adoption, depending on treatment regimen. One analysis modeled four strategies (label, PRN, treat-and-extend, and real-world evidence (RWE) regimen) over 1 year for a hypothetical 1-million-member health plan. Using the manufacturer-recommended regimen or real-world regimen, inclusion of brolucizumab yielded annual savings (~USD 31–32 million nationwide) [37], primarily via reduced injection costs. Even at the health-plan level, modest savings per member-month were seen. In contrast, PRN or fixed T&E regimens increased costs (because more injections were given). Overall, the studies suggest that under typical labeling or observed practice, brolucizumab can reduce the economic burden of nAMD.

For DME, cost-effectiveness is more tenuous. Brolucizumab’s competition includes off-label bevacizumab (extremely low cost) and aflibercept/ranibizumab. The Canadian Agency for Drugs and Technologies in Health (CADTH) reviewed brolucizumab for DME and noted high uncertainty. Their reanalysis (comparing brolucizumab vs. bevacizumab) yielded an incremental cost-effectiveness ratio (ICER) of ~USD 61,600 CAD per QALY (bevacizumab cheapest comparator) [38]. They concluded that, unless brolucizumab’s price is discounted substantially, it would not be cost-effective compared to bevacizumab (the lowest-cost therapy). Indeed, CADTH found no justification for a price premium of brolucizumab over other anti-VEGFs for DME, noting that equal-efficacy assumptions made brolucizumab more costly in their model. Thus, while brolucizumab may reduce treatment visits (and their indirect costs) in DME, formal cost-effectiveness hinges on price and choice of comparator. In payer decisions, agencies will likely factor in its clinical benefits against these economic considerations.

Brolucizumab represents an evolution of anti-VEGF therapy for retinal disease. Its unique structure and dosing regimen provide notable advantages: enhanced potency and the possibility of extended dosing intervals. The HAWK/HARRIER and KESTREL/KITE trials robustly demonstrated that brolucizumab yields vision improvements comparable to current standards while achieving better retinal drying and longer-lasting effects [11,24]. For busy patients and overburdened clinics, the prospect of going 12 weeks (or more) between injections is compelling. Real-world reports generally confirm that many eyes can indeed extend intervals and maintain vision [11,19]. However, these benefits are tempered by the safety profile. Brolucizumab’s IOI risk is higher than for ranibizumab or aflibercept [5]. Although the absolute rates are low (single-digit percentages), the potential for severe vision loss mandates caution. The mechanism likely involves immune complex formation given brolucizumab’s high immunogenicity [8]. Current evidence suggests the risk is greatest early in treatment [32], so clinicians are advised to examine patients frequently during the first 2–3 months and educate patients about symptoms. The MERLIN results have led to updated guidance: brolucizumab should not be dosed at <8-week intervals beyond loading [12]. Future work should clarify predictors of IOI (e.g., genetic markers, pre-existing antibodies) and optimal management strategies (role of local vs. systemic steroids).

Compared to novel anti-VEGF agents, brolucizumab holds its own. Faricimab (an Ang-2/VEGF bispecific) is another long-acting newcomer approved for nAMD and DME [39,40]. Indirectly, trials suggest similar efficacy [41]. Cost comparisons will be needed. At the least, brolucizumab expands choices, allowing personalized treatment. If an eye developed IOI on one agent, another may be tried (though cross-reactivity of inflammation risks is unknown).

Pediatric use remains largely uncharted. Brolucizumab is not indicated in children, due to lack of data. Rare reports (e.g., Coats’ case) [35] are hypothesis-generating but no substitute for trials. Pediatric retinal disorders are heterogeneous and rare; one might use brolucizumab only in extenuating cases where other therapies failed.

Pharmacokinetic and pharmacodynamic properties of brolucizumab (small size, high concentration) reliably translate to the clinical observations of potent VEGF suppression. The rapid systemic clearance and low serum exposure mean systemic side effects are unlikely, aligning with the neutral systemic safety data [8].

Economically, the balance appears favorable in nAMD when the cost of drugs and procedures are considered over time [36,37]. However, healthcare systems vary. In countries where aflibercept and ranibizumab are funded, brolucizumab’s higher price may be offset by fewer treatments. Where bevacizumab is the primary agent (e.g., many public systems), brolucizumab must compete on both efficacy and cost fronts.

Limitations of this review include reliance on published data to late 2024, with many ongoing studies. Particularly, ongoing surveillance of post-marketing events and new head-to-head trials (vs faricimab, etc.) will inform future use. Data on very long-term outcomes beyond 2 years are still emerging. The patient-level predictors of good response vs. risk are not well defined—an unmet need.

## 5. Conclusions

Brolucizumab has established itself as an effective treatment for nAMD and DME, expanding the anti-VEGF armamentarium with a highly potent, long-acting option. In clinical trials, it achieved vision gains on par with aflibercept while offering superior anatomical outcomes and the possibility of extending treatment intervals to 12 weeks or longer. Real-world experience corroborates these benefits in many patients. However, brolucizumab’s unique risk profile requires vigilance. The incidence of intraocular inflammation, including occlusive vasculitis, is higher than with earlier agents. Strict adherence to dosing recommendations (no sooner than q8w), careful monitoring, and early intervention for inflammation are critical to ensure safety.

In summary, for adult patients inadequately controlled by other anti-VEGFs or seeking reduced injection frequency, brolucizumab is a valuable option. Its use should be guided by a balanced discussion of its potential for fewer injections against its inflammation risks. Pharmacokinetic data support its dosing regimen, and cost analyses suggest health-economic advantages in many settings. Further research into optimizing safety (e.g., prophylactic strategies, biomarkers) and long-term outcomes will refine its role. Use in children remains investigational. Overall, the data indicate that with proper precautions, the efficacy advantages of brolucizumab make it a significant advancement in managing retinal neovascular diseases.

## Figures and Tables

**Table 1:** Search strings utilized across the databases.

Database	Full Strategy (Copy/Paste)
PubMed/MEDLINE	(“Brolucizumab” [Supplementary Concept] OR brolucizumab [tiab] OR Beovu [tiab]) AND ((“Macular Degeneration” [Mesh] OR “macular degeneration, age-related” [tiab] OR “age-related macular degeneration” [tiab] OR “neovascular age-related macular degeneration” [tiab] OR “wet AMD” [tiab]) OR (“Diabetic Macular Edema” [Mesh] OR “diabetic macular edema” [tiab] OR DME [tiab])) AND (safety [tiab] OR “intraocular inflammation” [tiab] OR “retinal vasculitis” [tiab] OR “retinal vascular occlusion” [tiab] OR “pharmacokinetics” [tiab] OR “pharmacodynamics” [tiab] OR “real-world” [tiab] OR “cost-effectiveness” [tiab] OR pediatric [tiab] OR effectiveness [tiab]) NOT (animals [mh] NOT humans [mh]) AND (english [lang])
Embase (Elsevier)	‘brolucizumab’/exp OR brolucizumab:ti,ab,kw OR beovu:ti,ab,kw AND ((‘age related macular degeneration’/exp OR ‘neovascular age related macular degeneration’:ti,ab,kw OR ‘wet AMD’:ti,ab,kw) OR (‘diabetic macular edema’/exp OR ‘diabetic macular oedema’:ti,ab,kw OR DME:ti,ab,kw)) AND (safety:ti,ab,kw OR ‘intraocular inflammation’:ti,ab,kw OR ‘retinal vasculitis’:ti,ab,kw OR ‘retinal vascular occlusion’:ti,ab,kw OR pharmacokinetics:ti,ab,kw OR pharmacodynamics:ti,ab,kw OR ‘real world’:ti,ab,kw OR ‘cost effectiveness’:ti,ab,kw OR pediatric:ti,ab,kw OR effectiveness:ti,ab,kw) AND [english]/lim AND [humans]/lim NOT ‘conference abstract’/it
Scopus (Elsevier)	TITLE-ABS-KEY (brolucizumab OR beovu) AND TITLE-ABS-KEY ((“age-related” W/3 macular W/3 degeneration) OR “neovascular age-related macular degeneration” OR “wet AMD” OR “diabetic macular edema” OR DME) AND TITLE-ABS-KEY (safety OR “intraocular inflammation” OR “retinal vasculitis” OR “retinal vascular occlusion” OR pharmacokinetics OR pharmacodynamics OR “real world” OR “cost effectiveness” OR pediatric OR effectiveness) AND (LIMIT-TO (LANGUAGE, “English”)) AND (EXCLUDE (DOCTYPE, “ch”))
Cochrane Library (CENTRAL & Reviews)	MeSH descriptor: [Brolucizumab] this term only OR brolucizumab:ti,ab,kw OR Beovu:ti,ab,kw AND (MeSH descriptor: [Macular Degeneration] explode all trees OR “neovascular age-related macular degeneration”:ti,ab,kw OR “wet AMD”:ti,ab,kw OR MeSH descriptor: [Diabetic Macular Edema] explode all trees OR “diabetic macular edema”:ti,ab,kw OR DME:ti,ab,kw) AND (safety:ti,ab,kw OR “intraocular inflammation”:ti,ab,kw OR “pharmacokinetics”:ti,ab,kw OR “pharmacodynamics”:ti,ab,kw OR “real world”:ti,ab,kw OR “cost effectiveness”:ti,ab,kw OR pediatric:ti,ab,kw) in Trials
Google Scholar	brolucizumab OR Beovu “age-related macular degeneration” OR “wet AMD” OR “diabetic macular edema” OR DME safety OR “intraocular inflammation” OR “retinal vasculitis” OR “retinal vascular occlusion” OR pharmacokinetics OR pharmacodynamics OR “real-world” OR pediatric OR “cost-effectiveness” filetype:pdf

**Table 2:** Demographic and baseline characteristics (Abbreviations: NR, not reported; RCT, randomized controlled trial; T&E, treat-and-extend; PRN, pro re nata; nAMD, neovascular age-related macular degeneration; DME, diabetic macular edema).

Study	Indication	Design	Region/Setting	Centers	N (Eyes/Patients)	Treatment Status	Follow-Up (Weeks)
HAWK [11]	nAMD	Phase III RCT, double-masked	Multinational	Multi	NR (part of combined *n* = 1817)	Naïve	96
HARRIER [11]	nAMD	Phase III RCT, double-masked	Multinational	Multi	NR (part of combined *n* = 1817)	Naïve	96
MERLIN [12]	nAMD with persistent fluid	RCT, head-to-head monthly	Multinational	Multi	NR	Refractory/persistent fluid	~100
SHIFT [20]	nAMD (refractory)	Observational, switch	NR (routine practice)	NR	NR	Refractory/switch	Short-term (post-1st dose)
Yeom et al. [21]	nAMD (recalcitrant)	Observational cohort, switch	Korea	Multi	81	Switch	~41 (mean) to 52
Inoda et al. [22]	nAMD	Observational T&E	Japan	Multi	107 eyes	Mixed (30 naïve, 77 switch)	52
BRAILLE [23]	nAMD	Observational PRN	India	Multi	94 eyes	Mixed (21.3% naïve; 78.7% switch)	52
KESTREL [24]	DME	Phase III RCT	Multinational	Multi	566	NR	100
KITE [24,25]	DME	Phase III RCT	Multinational	Multi	360	NR	100
KINGFISHER [26]	DME	RCT, monthly dosing	Multinational	Multi	517	Mixed (naïve/previously treated)	NR (~52)

**Table 3:** Technical and outcome features (abbreviations: AFL, aflibercept; Δ, change; VA, visual acuity; CST, central subfield thickness; IRF, intraretinal fluid; SRF, subretinal fluid; q4w/q8w/q12w, every 4/8/12 weeks; NA, not applicable; NR, not reported).

Study ID	Brolucizumab Dose	Loading Regimen	Maintenance Policy	Comparator	% on q12w at Key Time	BCVA Δ (Letters; Time)	CST Δ (µm; Time)	Fluid Outcome	Injection Burden/Interval	IOI (%)	Vasculitis/Occlusion (%)
HAWK [11]	3 mg and 6 mg	3 monthly	q12w, adjust to q8w per activity	Aflibercept 2 mg q8w	56% (W48)	+6.1 (3 mg), +6.6 (6 mg) vs. +6.8 (AFL), W48	−173 (6 mg) vs. −144 (AFL), W48	Persistent fluid lower at W16 (24.0% vs. 34.5%)	q12w maintained in majority eligible	NR	NR
HARRIER [11]	6 mg	3 monthly	q12w, adjust to q8w per activity	Aflibercept 2 mg q8w	51% (W48)	+6.9 (6 mg) vs. +7.6 (AFL), W48	−194 (6 mg) vs. −144 (AFL), W48	Persistent fluid lower at W16 (22.7% vs. 32.2%)	q12w maintained in majority eligible	NR	NR
MERLIN [12]	6 mg	Monthly start	Monthly (q4w)	Aflibercept monthly	NA (monthly)	Non-inferior BCVA to ~2 y	NR	Fluid-free 52.5% vs. 28.2% (W100)	q4w throughout	11.5 vs. 6.1	0.8/2.2 vs. 0/0.6
SHIFT [20]	6 mg (typical)	Single dose assessed	PRN post-switch	None (switch cohort)	NA	No significant short-term VA change	NR	SRF 57.1% resolved; IRF 40.7%; sub-RPE 20% after 1 dose	NR	NR	NR
Yeom et al. [21]	6 mg (switch)	Per clinic	T&E/extend if dry	None (switch cohort)	30.8% ≥q12w by 1 y	+6.6 (1 y)	−113 (*p* < 0.001)	83% “good responders” (fluid ↓ or interval ↑)	Interval extension common	9.9	~0/1.2 (1 occlusion)
Inoda et al. [22]	6 mg	Per clinic	T&E	None (mixed cohort)	~q12–13w at W52	Stable/improved	Stable/improved	Fluid control maintained	Final interval ~12–13w	NR	NR
BRAILLE [23]	6 mg	PRN with loading as needed	PRN	None (mixed cohort)	NR	Improved W4 → W52	CST ↓ markedly W12 → W52	SRF 78.05% → 21.95%; IRF 81.7% → 29.27% (W52)	4.8 ± 0.77 injections/52 w	NR	NR
KESTREL [24]	3 mg and 6 mg	5 q6w	q12w, adjust to q8w	Aflibercept q8w (after 5 q4w)	32.9% (BRO6, W100)	+8.8 (BRO6) vs. +10.6 (AFL), W100	Greater fluid resolution (NR for µm)	Fewer eyes with IRF/SRF vs. AFL	Fewer visits with q12w maintenance	NR	NR
KITE [24,25]	6 mg	5 q6w	q12w, adjust to q8w	Aflibercept q8w (after 5 q4w)	47.5% (BRO6, W100)	+10.9 (BRO6) vs. +8.4 (AFL), W100	Greater fluid resolution (NR for µm)	Fewer eyes with IRF/SRF vs. AFL	Fewer visits with q12w maintenance	NR	NR
KINGFISHER [26]	6 mg	Monthly start	Monthly (q4w)	Aflibercept monthly	NA (monthly)	+12.2 vs. +11.0 (Δ +1.1; 95% CI −0.6, 2.9)	−237.8 vs. −196.5 (*p* < 0.001)	Fluid-free 41.6% vs. 22.2% (*p* < 0.001)	q4w throughout	4.0 vs. 2.9	0.9/— vs. 0.6/—

## Data Availability

All data underlying this study are derived from publicly available journal articles.
